# Learned EEG-based brain self-regulation of motor-related oscillations during application of transcranial electric brain stimulation: feasibility and limitations

**DOI:** 10.3389/fnbeh.2014.00093

**Published:** 2014-03-18

**Authors:** Surjo R. Soekadar, Matthias Witkowski, Eliana G. Cossio, Niels Birbaumer, Leonardo G. Cohen

**Affiliations:** ^1^Human Cortical Physiology and Neurorehabilitation Section, NINDS, NIHBethesda, MD, USA; ^2^Applied Neurotechnology Lab, Department of Psychiatry and Psychotherapy, University Hospital TübingenTübingen, Germany; ^3^Institute of Medical Psychology and Behavioral Neurobiology, University of TübingenTübingen, Germany; ^4^Ospedale san Camillo, IRCCSVenice, Italy

**Keywords:** brain-machine interface (BMI) control, motor imagery, EEG, transcranial electric stimulation (TES), stimulation artifacts

## Abstract

**Objective:** Transcranial direct current stimulation (tDCS) improves motor learning and can affect emotional processing and attention. However, it is unclear whether learned electroencephalography (EEG)-based brain-machine interface (BMI) control during tDCS is feasible, how application of transcranial electric currents during BMI control would interfere with feature-extraction of physiological brain signals and how it affects brain control performance. Here we tested this combination and evaluated stimulation-dependent artifacts across different EEG frequencies and stability of motor imagery-based BMI control.

**Approach:** Ten healthy volunteers were invited to two BMI-sessions, each comprising two 60-trial blocks. During the trials, learned desynchronization of mu-rhythms (8–15 Hz) associated with motor imagery (MI) recorded over C4 was translated into online cursor movements on a computer screen. During block 2, either sham (session A) or anodal tDCS (session B) was applied at 1 mA with the stimulation electrode placed 1 cm anterior of C4.

**Main results:** tDCS was associated with a significant signal power increase in the lower frequencies most evident in the signal spectrum of the EEG channel closest to the stimulation electrode. Stimulation-dependent signal power increase exhibited a decay of 12 dB per decade, leaving frequencies above 9 Hz unaffected. Analysis of BMI control performance did not indicate a difference between blocks and tDCS conditions.

**Conclusion:** Application of tDCS during learned EEG-based self-regulation of brain oscillations above 9 Hz is feasible and safe, and might improve applicability of BMI systems.

## Introduction

Brain-machine interfaces (BMI) translate physiological features of brain activity associated with the user's intention or state into control signals of a particular device or computer (Birbaumer and Cohen, 2007). BMIs are increasingly used in the context of neurorehabilitation, e.g., after stroke (Ang et al., 2011; Ramos-Murguialday et al., 2013) with two main purposes: (1) to drive assistive devices or computers that surrogate a lost or impeded function, or (2) to facilitate motor recovery related to BMI-based neurofeedback training (Wang et al., 2010; Soekadar et al., 2011). The best-established BMI in stroke is based on volitional modulation of mu-rhythm (8–15 Hz, also called sensorimotor rhythm, SMR) recorded over the sensori-motor cortex using electro- or magnetoencephalography (EEG/MEG) (Buch et al., 2008; Ramos-Murguialday et al., 2013). Motor planning, imagery or execution is associated with a decrease in amplitude of mu-rhythm (Pfurtscheller and Neuper, 1997; Llanos et al., 2013). This decrease can be quantified as event-related desynchronization (ERD) (Pfurtscheller and Aranibar, 1979) and used by a BMI system to control an exoskeleton moving a patient's paralyzed hand (Soekadar et al., 2011).

Depending on the physiological features, several sessions of BMI training are typically required to achieve stable and reliable BMI control (Soekadar et al., 2011). Training-related BMI learning, however, is often substantially impeded in patients with brain lesions, such as stroke, traumatic brain injury or other brain disorders resulting in compromised learning capacity (Buch et al., 2008). Thus, it would be desirable to identify strategies to improve BMI learning and performance in these populations.

Several studies suggest that the application of weak electric currents in the form of transcranial direct current stimulation (tDCS) can improve motor learning (Zimerman et al., 2012), cognition (Metuki et al., 2012), memory consolidation (Dockery et al., 2009; Reis et al., 2009) and emotional processing (Nitsche et al., 2012). While the underlying physiological mechanisms of these effects are still unknown, it has been shown that tDCS can result in polarity-dependent shifts of membrane potentials and modulate cortical excitability (Nitsche and Paulus, 2000; Pellicciari et al., 2013). Recent work indicates that anodal tDCS applied before a motor imagery-based BMI session might improve BMI control (Wei et al., 2013) and modulate motor imagery-related mu-ERD (Matsumoto et al., 2010). A series of experiments suggests that learning is faster when tDCS is applied during a task compared to when it is applied prior to the task (Stagg et al., 2011). It was concluded that such timing-dependency might be of particular importance for the development of plasticity-inducing stimulation protocols (Nitsche et al., 2007). Furthermore, in addition to being time-saving in the clinical context when applied during motor or BMI training, direct effects of tDCS on brain oscillatory activity in the context of such training can be assessed, allowing for a better understanding of how transcranial electric currents modulate task-related brain activity.

While a novel strategy using magnetoencephalography (MEG) for *in vivo* assessment of neuromagnetic brain oscillations during transcranial electric brain stimulation was recently introduced (Soekadar et al., 2013a), the immobility and costs of MEG constrains broader clinical applications, e.g., for BMI training in the context of neurorehabilitation. EEG, in contrast, is inexpensive and widely used in clinical environments. However, the application of electric currents at voltages of up to 20 volts to the human head while recording EEG in the range of millivolts is particularly challenging. This is due to two main reasons: (1) Most EEG systems use wet electrodes to improve conduction between electrodes and the skin. Likewise, the application of electric currents to the head also requires good conduction, mostly provided by using electrolyte gels or pastes. If the conductive agents used for the EEG electrodes and stimulating electrodes are in direct contact, the stimulation currents will saturate the EEG amplifier and impede any physiological recordings. (2) Electric stimulation of the head can be associated with additional stimulation-depended signals picked up by the EEG amplifiers that might reduce classification accuracy in BMI control. This would be disadvantageous for both assistive and neurofeedback BMI applications. While denoting any stimulation-dependent signal during neurophysiological recordings of brain activity as artifacts or noise (i.e., an irregular fluctuation of the measured signal that does not contain meaningful information or obscures the information of interest) is somewhat appealing, it should be emphasized that the separation between the physiological responses or effects of the stimulation and stimulator-dependent noise is difficult. While recently systems were introduced that integrate electric stimulation and recording of EEG (e.g., Starstim® by Neuroelectrics, Barcelona, Spain) allowing for simultaneous EEG monitoring during tDCS (Schestatsky et al., 2013), it remained unclear how EEG signals across different frequency bands recorded at different distances from the stimulating electrode are affected by tDCS, and whether learned electroencephalography (EEG)-based brain-machine interface (BMI) control e.g., using motor imagery (MI) during tDCS is feasible, reliable, and safe.

**Figure 1:**
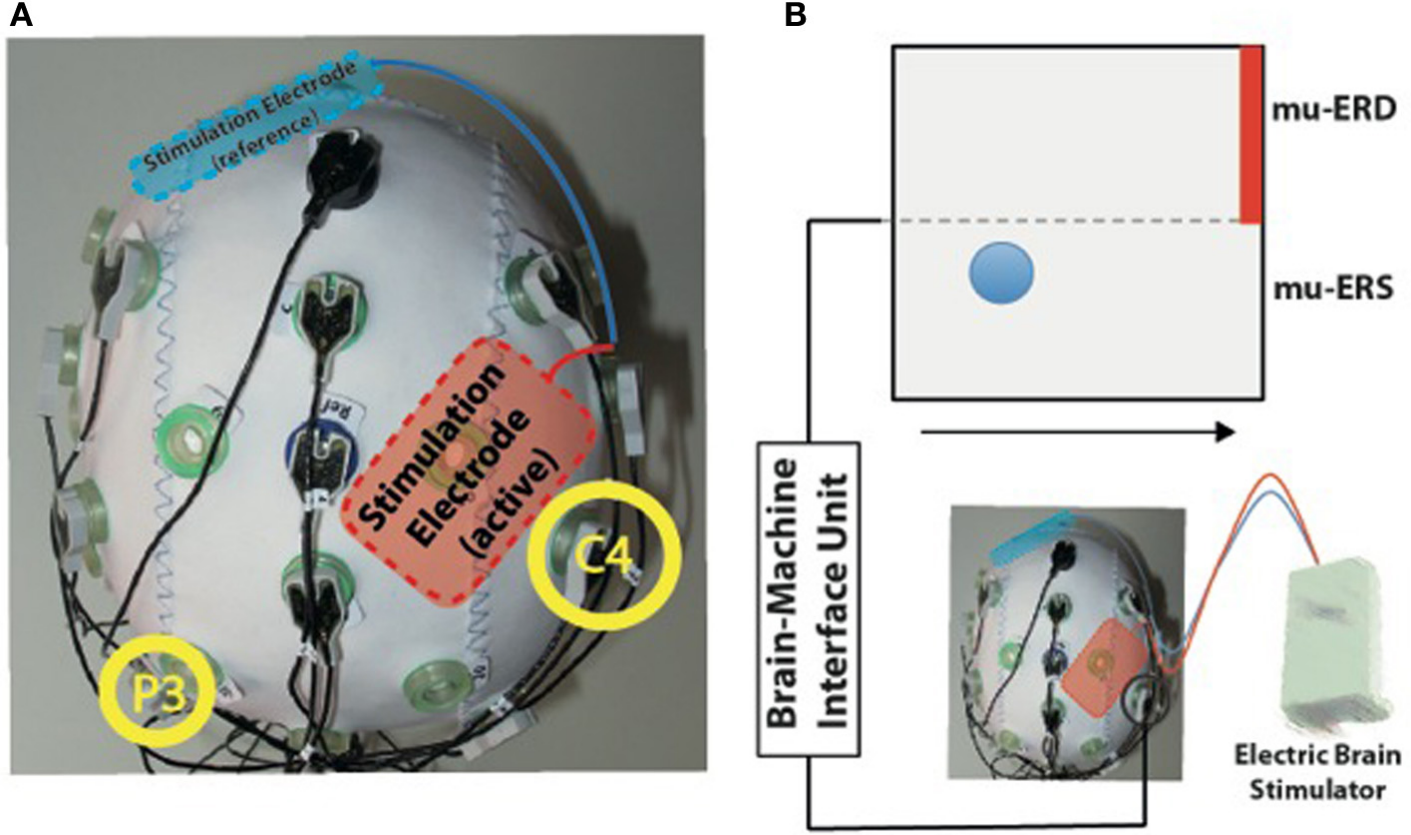
**(A)** Experimental setup for simultaneous transcranial electric current stimulation during electroencephalography (EEG). The active stimulation electrode was placed immediately anterior to the EEG electrode C4 used for online brain-machine interface (BMI) control (right yellow circle). The reference stimulation electrode was placed over the left supraorbital region (blue). **(B)** BMI paradigm. Electric brain activity recorded at electrode C4 was translated into visual feedback. Event-related desynchronization of mu-rhythms (9–15 Hz, mu-ERD) was indicated by upward movements of a blue ball, while mu-event-related synchronization was indicated by downward movements (mu-ERS). Participants were instructed to keep the ball above the dotted horizontal line during the task to hit the target (indicated by red bar).

Here, we investigated such a combination choosing an electrode montage that places the stimulation electrode as close as 1 cm to the EEG electrode recording brain oscillations used for BMI control (see Figure 1A). To allow generalization to other BMI paradigms, we first characterized stimulation-dependent signals at different EEG locations. We then compared the signal power in different frequencies (delta: 0.1–4 Hz; theta: 4–9 Hz; alpha: 9–15 Hz; and beta: 15–30 Hz) before stimulation and during stimulation, and identified those frequencies significantly influenced by stimulation-dependent signals. Finally, we calculated the effect size of the stimulation conditions for each frequency band. Potential side effects of stimulation, like tingling, itching or burning (Brunoni et al., 2011) were assessed throughout the sessions.

## Materials and methods

### Participants and experimental design

Ten healthy volunteers (5 males, 5 females, mean age: 26.7 years ± 4.3) exhibiting reliable and stable motor imagery-based BMI control in a previous investigation (Soekadar et al., 2011) were invited for two BMI sessions on consecutive days. All participants were right handed according to the Edinburgh Handedness Inventory (Oldfield, 1971). Each session was divided into two blocks of 60 trials (block 1, block 2). While no stimulation was delivered during block 1 of both sessions, either sham stimulation (session A) or anodal stimulation (session B) was applied during block 2. Sessions were conducted in random order. All participants gave written informed consent before entering the study. The study protocol was approved by the National Institute of Neurologic Disorders and Stroke Institutional Review Board (NINDS IRB).

### Electroencephalographic (EEG)-recordings and feedback of mu-event-related desynchronization (mu-ERD)

Participants were seated comfortably in an armchair facing a computer monitor. EEG was recorded from the following conventional EEG-recording sites (F3, FC5, C3, P3, Fz, AFz, Cz, FCz, F4, FC6, C4, P4 according to the international 10/20 system) using a 12-channel active electrode EEG system (Acti-cap® and BrainAmp MRplus®, BrainProducts, Gilching, Germany) with the reference electrode placed at FCz and the ground electrode at AFz. For translation of neurophysiological signals into visual feedback, BCI2000, a multipurpose standard BCI platform, was used (Schalk et al., 2004).

All participants were instructed to use visuo-kinesthetic MI of moving their left hand modulating right-hemispheric mu-rhythms when they saw the visual cue indicating the initialization of each trial.

To rule out overt movements during MI, electromyography (EMG) was recorded from the first dorsal interosseus muscle (FDI), extensor digitorum communis (EDC), extensor carpi ulnaris (ECU) and flexor carpi radialis (FCR) during the sessions. Skin/electrode resistance was kept below 10 kΩ. EMG signals were recorded at a sampling rate of 1 kHz and high-pass filtered at 2 Hz (BrainAmp ExG®, Brainproducts, Gilching, Germany). Trials in which EMG activity exceeded that recorded during rest by two standard deviations were interrupted and excluded from further analysis. The number of excluded trials in which EMG activity was present was comparable across participants and ranged between 5 and 10%.

Amplitude of mu-rhythm event-related desynchronization (mu-ERD) and synchronization (mu-ERS) was visually fed back to the participants during each trial (see Figure 1B). While increasing mu-ERD was indicated by up-movements of a ball above the horizontal midline of the screen, mu-ERS resulted in down-movements below the midline. Visual feedback was continuously updated every 100 ms. Computation of mu-ERD/mu-ERS included the power spectrum estimation (an autoregressive model of order 16 using the Yule–Walker algorithm) of each incoming sample at the optimal frequency for mu-ERD detection. The optimal frequency was identified in a screening run before the first session (11 Hz in all participants). Resulting values were compared with mean power values of the preceding inter-trial-intervals (ITI) that were continuously updated during BMI control according to the method of Pfurtscheller and Aranibar (1979) and as previously implemented into a mu-ERD BMI system (Soekadar et al., 2011):

(1) $$\bar{R}=\frac{1}{N}\sum_{t\;=\;1}^{N}R_{t}$$

(2) $$ERD\left(t\right)=\frac{T_{t}}{\bar{R}}-1$$

Where *t* represents the recorded sample block, *T*_*t*_ the event-related task condition period and *R*_*t*_ the power estimate in a given frequency band of *t*. *R* (reference value) represents power estimates during the rest (task-free) condition.

Each session consisted of 2 blocks with 60 trials. After the first block either sham stimulation (session A) or anodal stimulation (session B) was applied during the following 60 trials in a randomized order.

### Transcranial direct current stimulation (tDCS)

tDCS was applied via two conducting 4 × 6 cm rubber electrodes and attached to the participant's head using a conductive paste (Ten20®, D.O. Deaver, Aurora, CO, USA). The adhesive features of this paste prevented any sliding or dislocation of the electrodes during the attachment of the EEG cap. A bipolar electrode montage (right M1 and left supraorbital area) was used (see Figure 1A) to deliver a current of 1 mA (current density 0.04 mA/cm^2^; total charge 0.048 C/cm^2^ using the DC-STIMULATOR PLUS, neuroConn GmbH, Germany). During sham stimulation, the DC stimulator was set up to apply an anodal current for 15 s and—at the offset—decrease stimulation intensity in a ramp-like fashion, a method shown to achieve a good level of blinding (Gandiga et al., 2006). Prior to the first block of the first session, the M1 hand-area was localized in all participants based on the motor evoked potential (MEP) hotspot of the first digit's interosseus muscle (FDI) using transcranial magnetic stimulation (TMS). After the end of block 2 of sessions A and B, all participants rated possible discomfort, pain, tingling, itching or burning associated with the stimulation on visual analog scales (VAS) to assess safety and tolerability of tDCS during BMI control.

### Offline analysis

**Figure 2:**
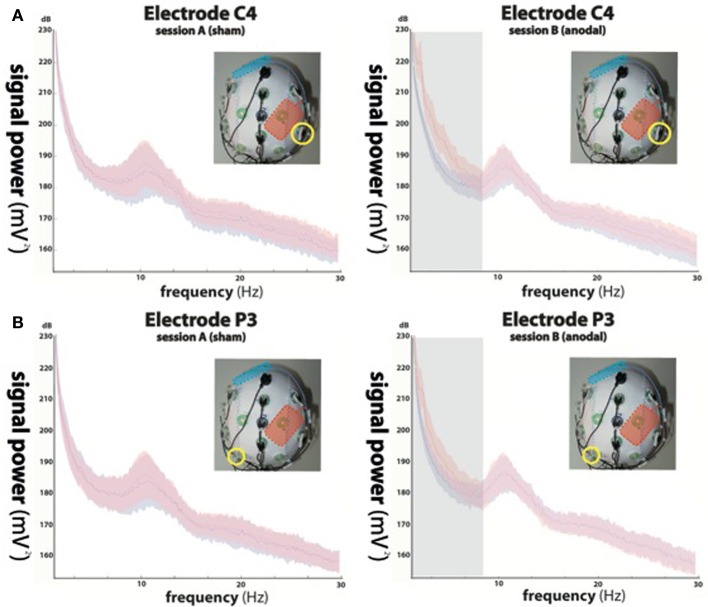
**Power spectra of the electroencephalographic (EEG) signals recorded from electrode C4 (A) indicated by yellow circles in the upper panels and P3 (B) during sham stimulation (left column, red curve) and anodal stimulation (right column, red curve)**. Power spectra of trials in absence of stimulation (block 1) are shown in blue. While not significant during sham stimulation (left column), anodal stimulation resulted in significant signal changes in delta (0–4 Hz) and theta (4–9 Hz) oscillations at electrode position C4 (indicated by the gray underlay in the upper right panel), equally present in delta, and in trend in theta oscillations recorded at P3 (indicated by the gray underlay in the lower right panel), while alpha (9–15 Hz) and beta (15–30 Hz) frequencies showed no difference between conditions.

For all outcome measures, assumption of a normal distribution (Shapiro–Wilk test of normality) was tested. Parametric tests were corrected by Greenhouse-Geisser estimates if Mauchly's sphericity test indicated significance. To compare signal power across conditions, fast Fourier transformations were performed for all EEG data collected during the first and second blocks of session A and session B (see Figure 2). A repeated-measures ANOVA (rmANOVA) with factors “block” (block 1, block 2) and “frequency” (delta, theta, alpha, beta) was performed based on the raw EEG signal power recorded from electrode C4 (in immediate proximity to the stimulation electrode) and P3 (at ~8–10 cm distance from the stimulation electrode) to investigate stimulation-dependent changes in different EEG channels. *Post-hoc* paired-samples Students *t*-tests were used when applicable and corrected for multiple comparisons (Bonferroni). Time-frequency representations (TFR) were plotted for both conditions and tested for statistical differences at two different electrode positions (C4 in immediate proximity of the stimulation electrode, and P3) using a cluster-based permutation test (Maris and Oostenveld, 2007). Effect size of stimulation-dependent signal differences in each frequency band was calculated using Cohen's *d* transformed into a regression coefficient *r* where *r* < 0.3 is considered a small, *r* < 0.5 is considered a medium, and *r* > 0.5 is considered a large effect (Cohen, 1988). A rmANOVA with factors “session” (session A, session B) and “block” (block 1, block 2) was used to evaluate changes of BMI control across sessions in the absence (block 1) and presence (block 2) of anodal tDCS. BMI control was defined as the time during each trial in which the ball was above midline (indicating mu-ERD). All analyses were performed in SPSS 17.0. Significance level was set to *p* < 0.05. Variance is defined as the standard error of the mean.

## Results

### Stimulation-dependent changes of signal power across different frequency bands and conditions

#### Stimulation electrode in close proximity (~1 cm) to the recording EEG channel (C4)

While rmANOVA of data recorded during session A (sham stimulation during block 2) from electrode C4 (Figure 2A) showed a main effect for “frequency” [*F*_(1, 27)_ = 37.156, *p* < 0.0001], indicating an expected difference between power values across the investigated frequency bands, there was no effect for “block” [*F*_(1, 27)_ = 0.233, *p* = 0.641] and no interaction between the factors (*p* = 0.188). *Post-hoc t*-tests showed no significant differences between power values of block 1 and 2 in any frequency band (delta: *p* = 0.729; theta: *p* = 0.963; alpha: *p* = 0.946; beta: *p* = 0.784).

When analyzing data of session B (anodal stimulation during block 2) from the same location, we found a main effect for both “frequency” [*F*_(1, 27)_ = 77.536, *p* < 0.0001] and “block” [*F*_(1, 27)_ = 33.16, *p* < 0.0001] and an interaction between the two [*F*_(1, 3)_ = 39.584, *p* < 0.0001]. *Post-hoc t*-tests showed a significant difference between power values of block 1 and 2 in the delta band (*p* < 0.0001) and a trend in the theta band (*p* = 0.068), but no significant differences in the alpha (*p* = 0.482) or beta (beta: *p* = 0.336) bands.

#### Stimulation electrode at ~8–10 cm distance to the recording EEG channel (P3)

Analysis of EEG data recorded ~8–10 cm away from the stimulation electrode (P3) (Figure 2B) during session A, revealed a main effect for “frequency” [*F*_(1, 27)_ = 28.28, *p* < 0.0001], but no effect for “block” [*F*_(1, 27)_ = 1.202, *p* = 0.301] and no interaction between the factors (*p* = 0.056). We found no significant differences between power values of block 1 and 2 in any frequency band (delta: *p* = 0.655; theta: *p* = 0.984; alpha: *p* = 0.937; beta: *p* = 0.923).

The same analysis performed for data acquired during session B (anodal stimulation during block 2) at electrode position P3, showed a main effect for both, “frequency” [*F*_(1, 27)_ = 34.39, *p* < 0.0001] and “block” [*F*_(1, 27)_ = 11.34, *p* < 0.01] and a significant interaction between the two [*F*_(1, 3)_ = 14.152, *p* < 0.0001]. *Post-hoc t*-tests indicated a significant difference between power values of block 1 and 2 in the delta band (*p* < 0.05), but not in the theta (*p* = 0.217), alpha (*p* = 0.445) or beta (beta: *p* = 0.482) bands.

**Figure 3:**
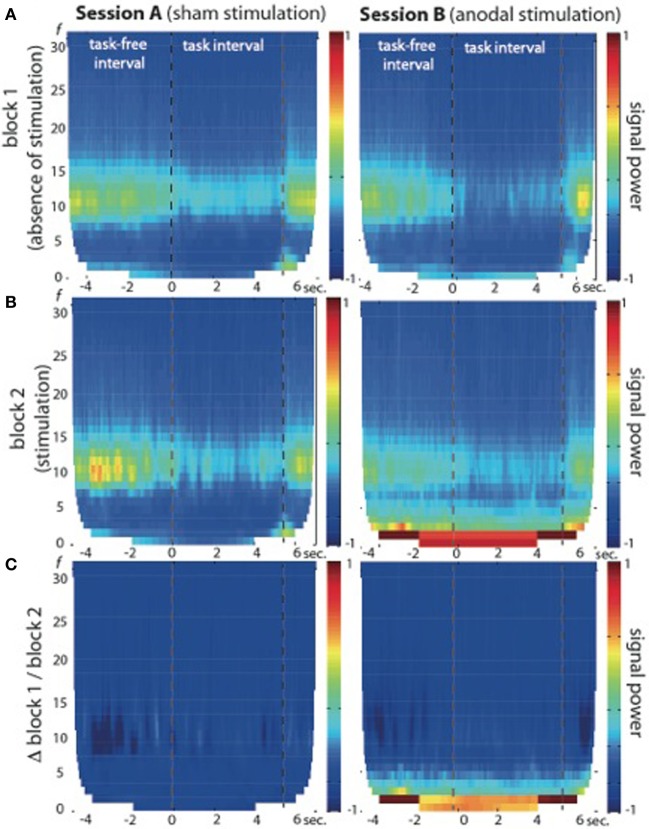
**Time-frequency representation (TFR) of brain oscillations recorded from EEG electrode position C4 at 1 cm distance from the active electric brain stimulation electrode. (A)** Block 1 of session A (left) and session B (right) in absence of electric brain stimulation. **(B)** Block 2 of session A (left, during sham stimulation) and session B (right, during anodal stimulation). Note the signal power increase in frequencies below 9 Hz in block 2 of session B (during anodal stimulation) across task-free and task intervals. **(C)** Signal power differences between block 1 and block 2 are plotted separately for both sessions (session A: left graph; session B: right graph), indicating no significant stimulation-dependent signal changes above 9 Hz.

Fourier transformations and TFR calculated for both sessions and blocks separately showed an increase in signal power during the second block of session B (see Figures 2, 3), which was highest in the slow frequency bands showing a decay of ~12 dB per decade. While not significant, we found a slight increase in broadband noise across all frequencies during anodal stimulation (second block, session B; see Figure 3B, right panel). Statistical analysis using a non-parametric cluster-based permutation test indicated significant stimulation-dependent changes of signal power in frequencies below 8 Hz (Figure 3C, right panel), but not in frequencies above 9 Hz. Calculation of the stimulation-dependent signal difference's effect size on each frequency band indicated a large effect on the delta band (*d* = 0.9893, *r* = 0.443) which was weaker in the theta band (*d* = 0.5721, *r* = 0.275) and small in the alpha (*d* = 0.2988, *r* = 0.122) as well as beta band (*d* = 0.2791, *r* = 0.115).

### Brain-machine interface (BMI) control across sessions and conditions

**Figure 4:**
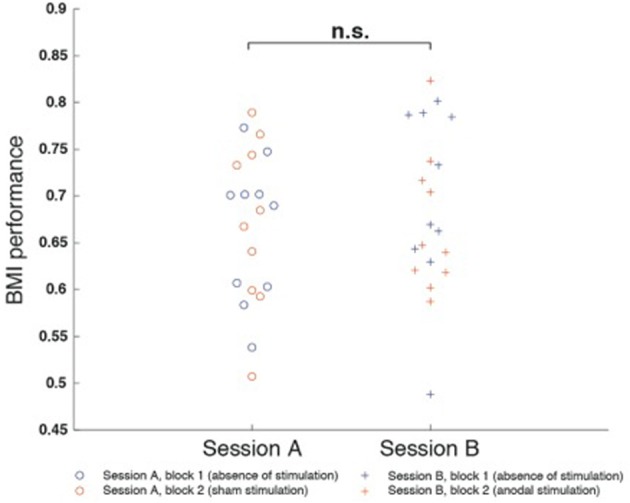
**Motor imagery (mu-event-related desynchronization, mu-ERD)-based brain-machine interface (BMI) performance was defined as the percentage of time mu-ERD was detected during trials**. BMI performance was comparable between both sessions and did not exhibit differences between block 1 and block 2. There was neither a difference between block 1 of session A and session B (*p* = 0.541), nor a difference between block 1 and block 2 of session A (*p* = 0.880) or session B (*p* = 0.470).

RmANOVA indicated no main effects for “session” [*F*_(1, 9)_ = 0.30, *p* = 0.597] or “block” [*F*_(1, 9)_ = 1.097, *p* = 0.322] and no interaction between the factors [*F*_(1, 1)_ = 0.349, *p* = 0.569]. There also was no difference between block 1 of session A and session B (*p* = 0.541), nor a difference between block 1 and block 2 of session A (*p* = 0.880) or session B (*p* = 0.470) (Figure 4).

### Safety and tolerability of simultaneous tDCS during EEG-based brain-machine interface (BMI) control

Six of ten participants reported light tingling (rated at 2–3 on a VAS, mean value across all participants 1.6 ± 0.4 with 0 = none and 10 = unbearable, extreme tingling) in at least one of the sessions, and five reported light itching (mean value 1.4 ± 0.8; 0 = none, 10 = unbearable, extreme itching), mainly at the beginning of block 2. None of the participants reported any form of severe discomfort or pain. Participants were unable to distinguish sensations between session A and session B.

## Discussion

Our study investigated the influence of simultaneous tDCS on EEG recordings across different frequency bands and shows that online extraction of physiological signals during learned self-regulation of brain oscillations for online MI-based BMI control is feasible and safe. We found that application of tDCS is associated with a significant signal increase across slower frequency bands below 9 Hz (delta and theta) in direct proximity of the stimulation electrode, and delta band (<4 Hz) recorded at larger distance (>~8 cm). However, signals oscillating above 9 Hz (e.g., 11 Hz) were not influenced by stimulation and could be successfully used for reliable, motor imagery-based BMI control. Our results indicate that any BMI paradigm driven by modulation of brain oscillations in the alpha (9–15 Hz) or beta range (15–30 Hz) or even higher frequencies (>30 Hz) is possible, given that the signal/noise ratio allows proper linear or non-linear classification of the neurophysiological features used for BMI control.

An important aspect in the combination of tDCS and BMI control is the stimulation montage. Due to the conduction properties of the human head, most electric currents pass through the skin and cerebro-spinal fluid (CSF), while only a fraction enters the gray or white matter (Sadleir et al., 2012). The path of the electric currents in any given case depends on many individual characteristics, such as bone thickness, shape of the skull, density of bone-passing veins or volume of the outer CSF space (which is increased, for instance, in brain atrophy). Various computational models were developed to calculate intracranial current flow and to identify brain areas with the highest magnitude of cortical electric fields (Bikson et al., 2012; Sadleir et al., 2012). Depending on the montage, the area with the highest magnitude of the cortical electrical field might be in larger distance from the stimulating electrode not directly underlying the current source (Edwards et al., 2013). Thus, the technique described here which allows for the application of electric currents as close as 1 cm to the EEG recording channel might be used for modulating cortical activity of brain areas functionally related to BMI control.

When using a different stimulator than the one used in this investigation, corresponding characteristics of this device should be tested first before combining it with EEG-based BMI systems to rule out other stimulation-dependent contaminations of the EEG signal.

While some previous studies suggested that tDCS can have immediate effects on motor imagery-related ERD (Matsumoto et al., 2010) and BMI control (Wei et al., 2013), we did not find such immediate effects. This might be due to the fact that participants were not BMI-naïve and exhibited already high and stable BMI control before admission to the study. tDCS, here applied as anodal tDCS, might not have improved BMI performance further, as a ceiling in BMI control may have been reached in these participants. Another reason could be the montage of the stimulation electrode placed 1 cm anterior of the C4, which might have resulted in the highest magnitude of cortical electrical fields in brain areas not related to motor imagery-based BMI control. A different placement of the stimulation electrode might have led to other results.

The immediate effects of electric currents on oscillatory activity in the human brain and their relatedness to behavior are still poorly understood (Dayan et al., 2013). As noted previously, a sensor-space based approach does not allow unambiguous differentiation between signals with a physiological origin opposed to stimulation-dependent signals deriving from the electric circuit of the stimulator. It is conceivable that the observed slow frequency signal power elevation during tDCS is in part of physiological origin and might reflect mechanisms that also underlie the previously well-described after-effects of tDCS, for instance polarity-specific modulation of cortical excitability and improvements of cognition and learning. Due to the design of the paradigm, the effect of tDCS on slow cortical potentials (SCP) could not be investigated here, but might be of interest in future studies to improve general understanding of the physiological effects of tDCS in the context of learned brain self-regulation during motor imagery-based BMI control. Implementation of algorithms with noise-canceling features, e.g., online source-reconstruction using beamformers (Soekadar et al., 2013b) might help to further investigate these mechanisms. The combination of electrical stimulation during multimodal EEG-MEG recordings in the context of brain self-regulation might help to shed light on these issues and further improve understanding of the exact neural substrates and mechanisms underlying the learning of abstract skills, like volitional modulation of brain oscillatory activity in the context of BMI control (Koralek et al., 2012).

## Conclusion

tDCS delivered at 1 mA in close proximity (1 cm) to an EEG channel used for learned self-regulation of brain oscillatory activity above 9 Hz is feasible and safe. While associated with a signal power elevation across slower frequencies, brain signals above 9 Hz were unaffected by the stimulation allowing simultaneous application of electric currents during motor imagery-based online BMI control. Such combination might substantially improve the applicability and practicality of BMI use in patient populations, for instance, in the context of neurorehabilitation, and allow systematic investigation of the relatedness between learned brain self-regulation, brain oscillatory activity and behavior.

### Conflict of interest statement

The authors declare that the research was conducted in the absence of any commercial or financial relationships that could be construed as a potential conflict of interest.
